# 1 vs 3 days laparoscopic suturing courses: is it feasible to design a valid training curriculum?

**Published:** 2020-10-08

**Authors:** IM Argay, T Lawrence, K Afors, G Centini, L Lazzeri, N Habib, N Amoruso, E Zupi, R Campo, A Wattiez

**Affiliations:** University of Debrecen Clinical Center, Department of Obstetrics and Gynaecology, H-4032 Debrecen, Nagyerdei Krt. 98, Hungary; Department of Obstetrics and Gynaecology, Whittington Hospital, London, United Kingdom; Department of Molecular and Developmental medicine, University of Siena, Siena, Italy; Obstetrics and Gynaecology Service, Beaujon Teaching Hospital, Clichy and Paris Diderot University, Clichy, France; Life Expert Centre, Tiensevest 168, 3000 Leuven, Belgium; Latifa Hospital, Dubai, United Arab Emirates.

**Keywords:** training, psychomotor skills, laparoscopy, suturing course

## Abstract

**Background:**

Laparoscopic skills are unlikely to be achieved exclusively in the operating theatre, so simulation training has become mandatory to acquire specific psychomotor skills to be merged in a more complex procedure.

**Objective:**

To compare 3-day vs. 1-day laparoscopic suturing courses and to better address participants’ needs according to their level of experience.

**Methods:**

Observational cohort study conducted between January 2017 and December 2018 including 107 participants amongst which 61 attended a 3-day and 46 the 1-day suturing course.

**Results:**

Data analysis showed no significant difference in the pre-test suturing scores between the two groups. On each course, when comparing the pre- and post-tests results, the participants reached a statistically significant improvement in both precision and knotting score (p< 0.01). However, when comparing the two types of courses, the data showed a better performance in the post-session test for those attending the 3-day course (p<0.05), as well as a higher mean score improvement (4.7 vs. 2.8; p<0.05) and time needed to complete exercises (-270s vs. -150s; p<0.05). Furthermore, grouping the participants according to their experience, the experts achieved a significantly better improvement attending the 3-day course, when compared to the beginners.

**Conclusions:**

Both 3 and 1-day course are successful in improving laparoscopic suturing skills regardless of the participant’s experience. However experienced participants benefit more from a longer course while the 1-day one should be dedicated to pre-surgical competences acquisition.

## Introduction

Gynaecological surgery is a significant field, with 1 in 7 women undergoing some form of gynaecological surgery in their lifetime. Minimally invasive (MIS) approaches to pelvic gynaecological surgery include both laparoscopic and robotic techniques, in addition to traditional open and vaginal approaches. Minimally invasive methods have been shown to shorten hospital admissions, decrease blood loss and infection rates, allowing for faster recovery and improved patient satisfaction when compared with open surgery (Agha and Muir, 2003).

The first laparoscopic assisted hysterectomy was performed in 1988, and since then, there has been an acceleration in the use of minimally invasive methods to carry out pelvic and abdominal surgery. An observational multicentric Dutch study in 2015 showed an increasing percentage of procedures performed by MIS, especially total laparoscopic hysterectomy. Indeed, the percentage of TLH increased significantly from 3% in 2002 to 10% in 2007 to 36% in 2012, whereas abdominal and vaginal hysterectomy decreased significantly, from 68% to 39% and from 29% to 25% respectively (Driessen et al., 2015).

With the advent of minimally invasive surgery, budding gynaecological surgeons are challenged with achieving competency using several different approaches and techniques. The learning curve for laparoscopic surgery is notoriously steep for a multitude of reasons; small incision, reduced tactile feedback, loss of depth perception and reduced number of instruments. Movements are often counterintuitive, because instruments should be used as a lever through fixed ports where the abdominal wall works as a fulcrum, challenging the novice in mastering a good angulation of the tissues.

Laparoscopic suturing and knot tying are some of the hardest skills to master and is still the major obstacle to the development of laparoscopy. Simulation training has been shown to improve skills and boost confidence in laparoscopic techniques by initiating the learning curve outside of the pressure of the operating theatre. In addition, health systems may demand that surgeons become certified in laparoscopy to avoid medicolegal litigation (Torres-de la Roche et al., 2019).

As reported in the literature a minimum of 30 procedures is required to reach the plateau phase of the learning curve for laparoscopic hysterectomy, in order to lower the rate of intraoperative complications and operating time (Ghomi et al., 2007). However, the increasing number of trainees, as well as alternatives to surgery reduces the ratio of exposure to laparoscopy. Given these challenges of acquiring the required skills, and the ever- diminishing volume of cases, competency is unlikely to be achieved solely in the operating theatre.

Therefore, to tackle these shortcomings and to decrease complications associated with the learning curve when undertaking practical procedures, simulation training has become increasingly popular, and a standardised training and assessment must be provided for surgeons (Torres-de la Roche et al, 2019).

This study primarily sought to compare the structure of two different courses for teaching laparoscopic suturing. The study’s secondary aim was to assess which course was more beneficial according to the experience of the participant, in order to optimise their training.

## Materials and methods

Data was prospectively collected from participants attending the laparoscopic suturing course held by the European Academy of Gynaecological Endoscopy based in Leuven, Belgium, between January 2017 and December 2018. A total of 5 courses were run. Four of these courses were 3 days in length, whilst the other was a single day course.

**Figure 1:**
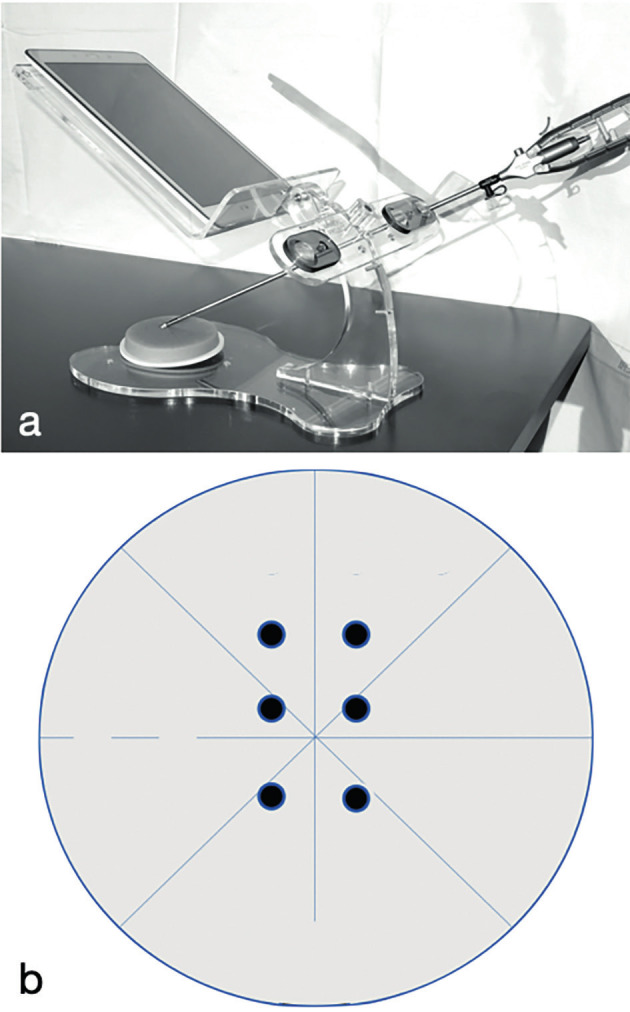
— a) the figure shows the pelvic trainer used for the 1 day course; b) example of the exercise performed by the participants as pre and post-test.

Each 3-day course was run with a maximum of 20 participants training in pairs on a Szabo-Berci pelvic trainer, with a Storz Telepack (Karl Storz, Tubingen, Germany) and a 0-degree 10 mm scope. The 1-day course was held with the participants training individually on a specific device designed for the course (Figure 1a). This device had two side roller-balls, with a 5 mm hole to simulate the trocars, and a L-shaped transparent support where a smartphone or tablet can be placed as a video/ light source. In each course the participants trained with two needle holders with curved tip and straight handle using a half circumference 26 mm needle with a braided 2-0 thread.

In both courses, the participants were required to fill in a form describing their previous laparoscopic experience. Then, they underwent a pre-course test prior to any didactic or practical training. For this pre-test, the participants were asked to perform 3 stitches across a vertical line on a sponge pad 6 cm in diameter and 8 mm in height, where the entry and exit points were marked by black dots of 4 mm in diameter 2 cm apart one each other as schematized in Figure 1b. Each stitch was tied with a locking sequence knot, and the exercise had to be completed in 15 minutes.

The form and the pre-course test were used to group the participants according to their experience, in categories of ‘expert’ and ‘beginners’. To be classified as an expert the participant had to score higher than 5 in the pre-test, with at least a complete knot with locking sequence, as well as fulfil one of the following criteria:

1) performed at least 50 laparoscopic procedure including 15 that require suturing such as myomectomy, sacropexy or hysterectomy with laparoscopic closure of the vaginal vault.

2) performed at least 30 laparoscopic procedure including 10 that require suturing such as myomectomy, sacropexy or hysterectomy with laparoscopic closure of the vaginal vault and 1 suturing course.

The participants underwent a step-by-step training programme, where each theoretical lesson was followed by a practical session. These sessions included the basic principles of laparoscopic suturing, needle loading, continuous running sutures, and knot tying using the gladiator technique and alternative knotting techniques (Asencio et al., 2018).

All courses were run by a group of tutors specifically trained to mentor the participants.. All tutors had attended at least five courses as a mentor before being enrolled. A constant ratio of 1 mentor for every 4 participants was maintained in all courses.

Once the training course was complete, the practical test was repeated. The practical post-course test was evaluated using the precision of the entry and exit of the suture in the dots, and the knot quality. The scoring system ranged from 0 to 12. Precision was assessed by awarding one point for each dot passed through with the stitch. The knot quality assessed for security of the locking of the knot and the quality of the tissue approximation. If precision and safety were achieved, the participant was awarded 2 points. If the knot was secure but not tight, the participant was awarded 1 point.

## Results

### Description of the participants

During the study period, 123 participants attended the course. 70 participants attended the 3-day course, and 53 the 1-day course. 16 participants were excluded from the data analysis because of missing information on the registration form or because the pre/post-course test was not performed. Therefore, 107 participants were included in the study, with 61 attending the 3-day and 46 the 1-day course.

**Figure 2:**
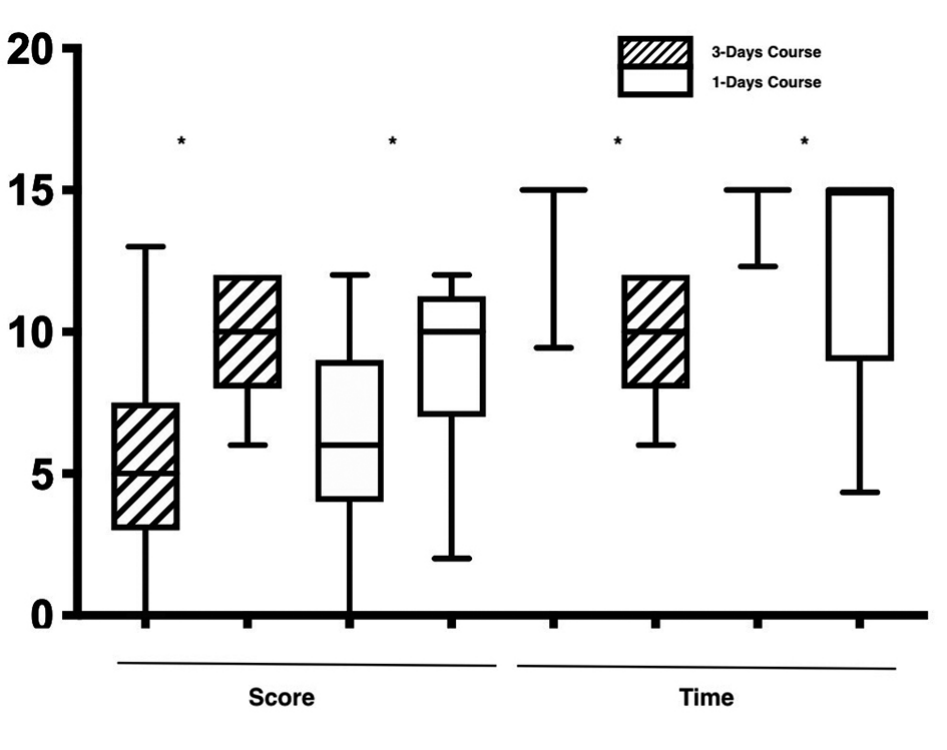
— a) The graph shows the score and time of the participants in the pre and post-course test (* p<0.05).

The data analysis shows a significant difference in suturing scores and timing comparing the pre and post-course test in both groups (p<0.001) (Figure 2).

When comparing the 2 courses there was no significant difference between the pre-course test. There was a better performance in the post-course test in favour of the 3 day course (P<0.05), as well as a higher mean score improvement of 4.7±3.0 vs. 2.8±3.5 (p<0.05). Similar results were found when comparing the time taken in the post-course test between the 3 and 1 day course (270s ± 216 vs. -150s± 216 p<0.05).

Comparing the data in the post-course test, 83% (51/61) of participants in the 3-day course and 43.5% (20/46) of participants in the 1-day course completed the exercise within 15 minutes. Amongst those, 37.7 % (23/61) of participants in the 3-day course and 19.5% (9/46) of participants in the 1-day course completed the exercise with a perfect score, showing the significant benefits of the 3-day course in terms of precision and knot tying overall (p<0.001).

When comparing the pre- and post-course test results, the 1-day course showed a statistically significant improvement in both precision, with a score of 3.1 ± 2.0 vs. 4.6 ± 1.8 (p<0.001) and knot tying, with a score 3.0 ± 2.1 vs. 4.2 ± 1.9(p=0.01). This was also true for the 3 day course, with suturing precision scores of 3.0 ± 1.6 vs. 5.0 ± 1.0 (p<0.001) and knot tying scores of 2.3 ± 1.6 vs. 5.0 ± 1.1 (p<0.001).

Comparing the suturing precision and knot tying scores within both groups, while the 1-day course showed no significant difference between precision and knot tying score, those attending the 3 day course showed a significant improvement in the knot tying, with scores of 1.9 ± 1.7 vs. 2.7 ± 1.8 (p=0.01).

When improvements in the suturing scores for the precision and the knot tying were compared between the groups, there was significant improvement in knot tying in the 3-day course group with suturing scores of 1.3 ± 3.0 vs. 2.7 ± 1.8(p<0.001). However, there was no significant difference when comparing the 2 groups in terms of suturing precision scores - 1.5 ± 2.1 vs. 1.9 ± 1.7 (p>0.05).

The groups were also evaluated based on their laparoscopic experience. The groups were divided into expert and non-expert based on the form completed at registration, and the time and score compared accordingly. The data, despite a higher score in the expert group, did not show any significant difference in the 1-day group.

In the 3-day course, the more experienced participants reported a higher score in the pre-course test, something that was maintained in the post- course test, achieving an overall higher score (10.9 ± 1.8 vs. 9.7±1.8) (p<0.05) and faster completion times (8.3 ± 3.3 vs. 10.3 ± 3.8 minutes) (p<0.05).

**Figure 3:**
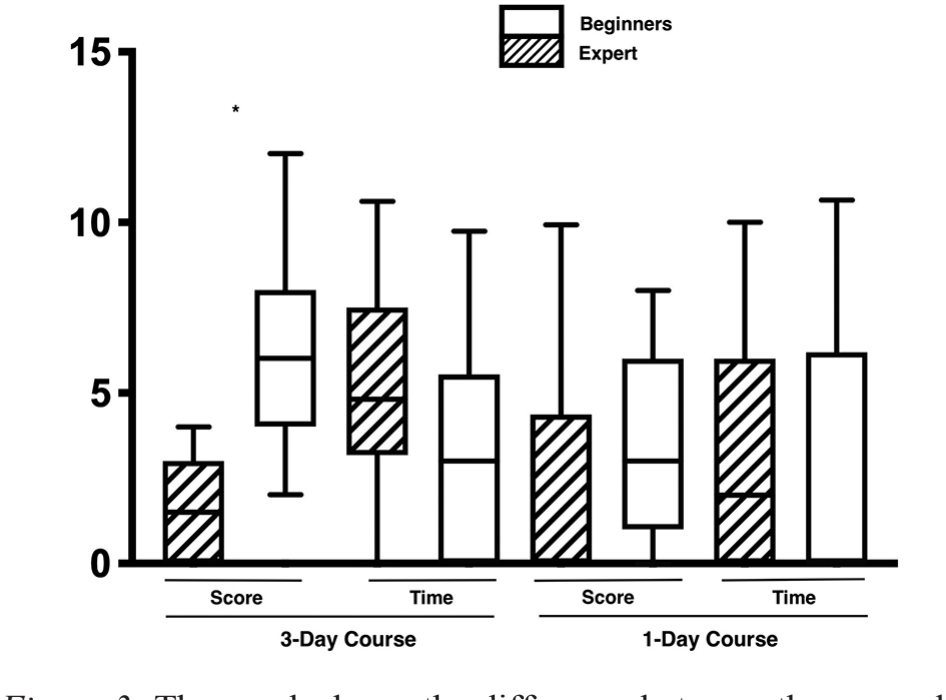
— The graph shows the difference between the pre and post-course test between the expert and the beginners (*p<0.05).

Furthermore, when the difference between the pre and post-course tests were calculated for both timing and suturing scores, the 1-day course did not show any difference, while the 3-day course reported a shorter completion time for the expert group, and a significant score improvement for the beginners (p<0.001) (Figure 3).

Additionally, the more experienced participants were more likely to finish the test on time with a full score. Indeed, in the expert group the 51.2% (20/39) who finished on time achieved a perfect score, while only 28.1% (9/32) of the beginners did (p<0.05).

Comparing beginners and experts across both groups reported a significant score improvement in favour of the 3 day course, while timing was significant only in the expert group (p<0.05).

## Discussion

This study sought to evaluate the benefit of a 1-day versus a 3-day course in laparoscopic suturing. Both groups had a similar skill mix and no significant difference in their pre-course suturing scores. After undergoing training in the theory and practice of laparoscopic suturing, both groups showed significant improvements in their overall suturing scores within the domains of both precision and knot tying. This confirms that the simulation course improved their skills, as expected.

Interestingly, those completing the 3-day course had significantly higher suturing scores overall in the post-course test, and a higher improvement in their suturing scores. A larger proportion of participants attending the 3-day course were able to finish the task within 15 minutes and achieved a perfect score when compared to the 1-day course group. It is generally accepted worldwide that loading the needle is the most challenging and time-consuming step during laparoscopic suturing. It was therefore not surprising that the participants in the 3-day course, who had significantly more time to hone their practical skills loading the needle, were able to complete the given tasks in less time than those in the 1-day course.

There was no significant difference in precision improvement between the two groups, however the 3-day course group had a greater improvement in their knot tying suturing scores. This can be easily explained; while the duration required for the theoretical component of the courses was relatively static, the 3-day course allows for more practice.

Successful laparoscopic suturing necessitates familiarity with several steps that require fine motor skills, hand-eye-video coordination, and the ability to overcome a lack of depth perception. It is not surprising that those who were able to benefit from more practice and expert advice due to the duration of the course were able to achieve higher suturing scores. Intracorporeal knot tying is difficult, requiring delicate, fine motion. Dedicating more time to the mastery of this skill in the 3-day course was reflected in the superior improvements in suturing scores when compared to the 1-day course. Precision improved overall in both groups, but there was no difference in the final suturing scores. Precision is a measure of overall suturing abilities and depth perception, and depends even more on the mastery of fine motor skills when compared to loading of the needle and knot tying. This skill requires more practice than can be afforded in a course setting and would likely improve with experience.

In both groups, those participants with more laparoscopic experience were more likely to complete the tasks within allotted time and achieve a perfect score. However, the novice group achieved greater improvement in suturing scores when comparing pre- and post-course test outcomes.

The more experienced group are likely to be further along the learning curve with laparoscopy, and more familiar with laparoscopic challenges such as lack of depth perception and the fulcrum effect of ports. This allows them to focus on the task of suturing, whilst the less experienced participants were faced with the above challenges, in addition to those of suturing. The less experienced participants, therefore, achieved the greatest improvement while the experts were more likely to perform a good quality knot. This indicates that, despite the challenges faced by novice laparoscopists, with didactic teaching, practical sessions and expert guidance, the difficulties of laparoscopy and suturing can be overcome, and the learning curve shortened.

As laparoscopy becomes increasingly part of our routine gynaecological practice, standardised training programmes are becoming a necessity, and laparoscopic suturing should be part of it. The European Academy of Gynaecological Surgery and European Society for Gynaecological Endoscopy (ESGE) strongly advocate a surgeon’s attendance of approved simulation programmes (Campo et al., 2016;Tanos et al., 2016;Ferreira et al., 2018;Sleiman et al., 2015). The Gynaecological Endoscopic Surgical Education and Assessment (GESEA) programme is the official diploma of ESGE that trains and certifies knowledge and practical skills in gynaecological endoscopy. It consists of three different practical tests of endoscopic surgery: LASTT, focused on basic laparoscopic psychomotor skills, HYSTT, based on basic hysteroscopic psychomotor skills in the uterine environment, and SUTT, focused on ability of fine and complex motor skills by performing stitches and intra-corporeal knots.

The last module consists of several exercises of increasing difficulty, divided into two levels, that measure ability of stitching, knot tying with both hands and tissue approximation. Therefore, one- day or three-day courses could help to shorten time needed to accomplish the official diploma.

Recently, Edler et al. (2017) reported a significant drop in hysterectomies of about 25% in Austria between 2002 and 2014 . This decrease in volume affects the ability of residents to become comfortable in performing major gynaecological surgeries. A recent review by Torres-de la Roche et al. (2019) found that only 58% of graduating residents were “completely prepared” to perform an abdominal hysterectomy, with only 28% for vaginal, 22% for laparoscopic, and 3% for robotic hysterectomy (Ghomi et al., 2007).

Mastering the challenges associated with laparoscopy requires dedicated time, practice, and can therefore be costly to run and attend. It is therefore important to balance the benefits of training opportunities with time.

Exposure time plays a pivotal role, and this is clearly demonstrated by a significant score improvement in favour of the 3-day course for both beginners and experts.

However, the 1-day course was still able to produce impressive improvements in each of the investigated categories. Therefore, we can appreciate that a shorter course would be an excellent option to train larger numbers of trainees in a more efficient manner.

Furthermore, our data shows that the improvement is different when comparing experts versus beginners. The difference between the two groups increases in the 3- day course while it is not significant in the shorter one. These findings suggest that exposure to training should be timed to optimise results at the beginning of a surgeon’s training. Generally speaking, is much better to start well than correct a rooted mistake later on.

Last but not least, the 51% of the experts who completed the exercise achieved a perfect score while in the beginner group only 28% did so. This indicates that despite the test being completed within the allocated time, the mentor found an error either in terms of precision or in the quality of the knots, suggesting that the experts were better at self-evaluating their work .

This is an important finding, not only for suturing but also in surgery, because it proves the role of experience as a part of the whole training programme; not only is it necessary to have the skills and to know how to perform a procedure, but it is important to be mentored to reduce complications.

The results of this study highlight the pivotal role that simulation plays in the training of surgeons and its standardisation. In this way, dry lab training can be merged with mentorship in the operating theatre in order to mitigate the risks associated with novice surgeons experiencing the challenges of laparoscopy in vivo.

## Limitations

There is a surprising paucity of data on the subject of simulation training for laparoscopic suturing in particular, and most of the research was done using participants with very minimal exposure to laparoscopy or with general surgeons rather that gynaecologists. Also, the 1-day course group used different equipment compared to the 3-day group. Although in theory the challenges associated with laparoscopy should have been simulated in a similar manner by both sets of equipment, this may have affected the outcomes with regards to suturing scores.

## Conclusion

In conclusion, this study clearly demonstrated that not only there is a benefit of simulation training for laparoscopic surgeons, regardless of their level of experience, but also that training should tailored to the participants.

A short course should be used to expose a larger number of less experienced trainees to the challenges associated with laparoscopic surgery prior to the operating room, while the longer 3-day course seems to be more suitable for more experienced surgeons as the extended practice time allowed for the refinement of accuracy.

## References

[1] Agha R, Muir G (2003). Does laparoscopic surgery spell the end of the open surgeon?. J R Soc Med.

[2] Asencio FA, Ribeiro HASA, Romeo A (2018). The effect on performance time and quality of the knots after mono or bimanual training of laparoscopic intracorporeal knot tying according to the gladiator rule technique.. Rev Bras Ginecol Obstet.

[3] Campo R, Wattiez A, Tanos V (2016). Gynaecological endoscopic surgical education and assessment. A diploma programme in gynaecological endoscopic surgery.. Gynecol Surg.

[4] Driessen SR, Baden NL, van Zwet EW (2015). Trends in the implementation of advanced minimally invasive gynecologic surgical procedures in the Netherlands.. J Minim Invasive Gynecol.

[5] Edler KM, Tamussino K, Fülöp G (2017). Rates and routes of hysterectomy for benign indications in Austria 2002–2014.. Geburtsh Frauenheilk.

[6] Ferreira H, van Belle Y, Tanos V (2018). Simulation and training of gynaecological skills.. Facts Views Vis Obgyn.

[7] Ghomi A, Littman P, Prasad A (2007). Assessing the learning curve for laparoscopic supracervical hysterectomy.. JSLS.

[8] Sleiman Z, Tanos V, Van Belle Y (2015). The European Academy laparoscopic "Suturing Training and Testing" (SUTT) significantly improves surgeons’ performance.. Facts Views Vis Obgyn.

[9] Tanos V, Socolov R, Demetriou P (2016). Implementation of minimal invasive gynaecological surgery certification will challenge gynaecologists with new legal and ethical issues.. Facts Views Vis Obgyn.

[10] Torres-de la Roche L, Leicher L, Steljes I (2019). Training and qualification in gynaecological minimal Access surgery: A systematic review. Best Pract Res Clin Obstet Gynaecol.

